# Persistence of coastal upwelling after a plunge in upwelling-favourable wind

**DOI:** 10.1038/s41598-020-67785-x

**Published:** 2020-07-20

**Authors:** Jihun Jung, Yang-Ki Cho

**Affiliations:** School of Earth and Environmental Sciences/Research Institute of Oceanography, Seoul National University, Seoul, 151-742 Republic of Korea

**Keywords:** Ocean sciences, Physical oceanography

## Abstract

Unprecedented coastal upwelling off the southern coast of the Korean Peninsula was reported during the summer of 2013. The upwelling continued for more than a month after a plunge in upwelling-favourable winds and had serious impacts on fisheries. This is a rare phenomenon, as most coastal upwelling events relax a few days after the wind weakens. In this study, observational data and numerical modelling results were analysed to investigate the cause of the upwelling and the reason behind it being sustained for such an extended period. Coastal upwelling was induced by an upwelling-favourable wind in July, resulting in the dynamic uplift of deep, cold water. The dynamic uplift decreased the steric sea level in the coastal region. The sea level difference between the coastal and offshore regions produced an intensified cross-shore pressure gradient that enhanced the surface geostrophic current along the coast. The strong surface current maintained the dynamic uplift due to geostrophic equilibrium. This positive feedback between the dynamic uplift and geostrophic adjustment sustained the coastal upwelling for a month following a plunge in the upwelling-favourable wind.

## Introduction

Coastal upwelling is a process that brings deep, cold water to the ocean surface. It can play an important role both in physical processes and in chemical and biological variability in coastal regions by transporting nutrients to the surface layer. Coastal upwelling can be induced by various mechanisms, but it generally results from Ekman transport due to the alongshore wind stress^1,2^. Wind stress curl can also induce coastal upwelling^3^. The current along a coastal region may enhance the onshore Ekman pumping through the bottom boundary layer^4,5^. Upwelling occurs as a form of dynamic (isotherm) uplift that results from geostrophic equilibrium, which is a balance between the pressure gradient force (PGF) and the Coriolis force^6,7^.

The southern sea region off the Korean Peninsula connects the East China Sea and the East/Japan Sea. The mean depth of this offshore region is approximately 100 m. There is an eastward alongshore flow throughout the year^8,9^. A two-layer structure, comprising warm water in the upper layer and cold water in the lower layer, forms during the summer. The surface waters that originate from the Kuroshio and East China Sea are heated by the atmosphere. The deep, cold water originates from the west^10,11^.

**Figure 1:**
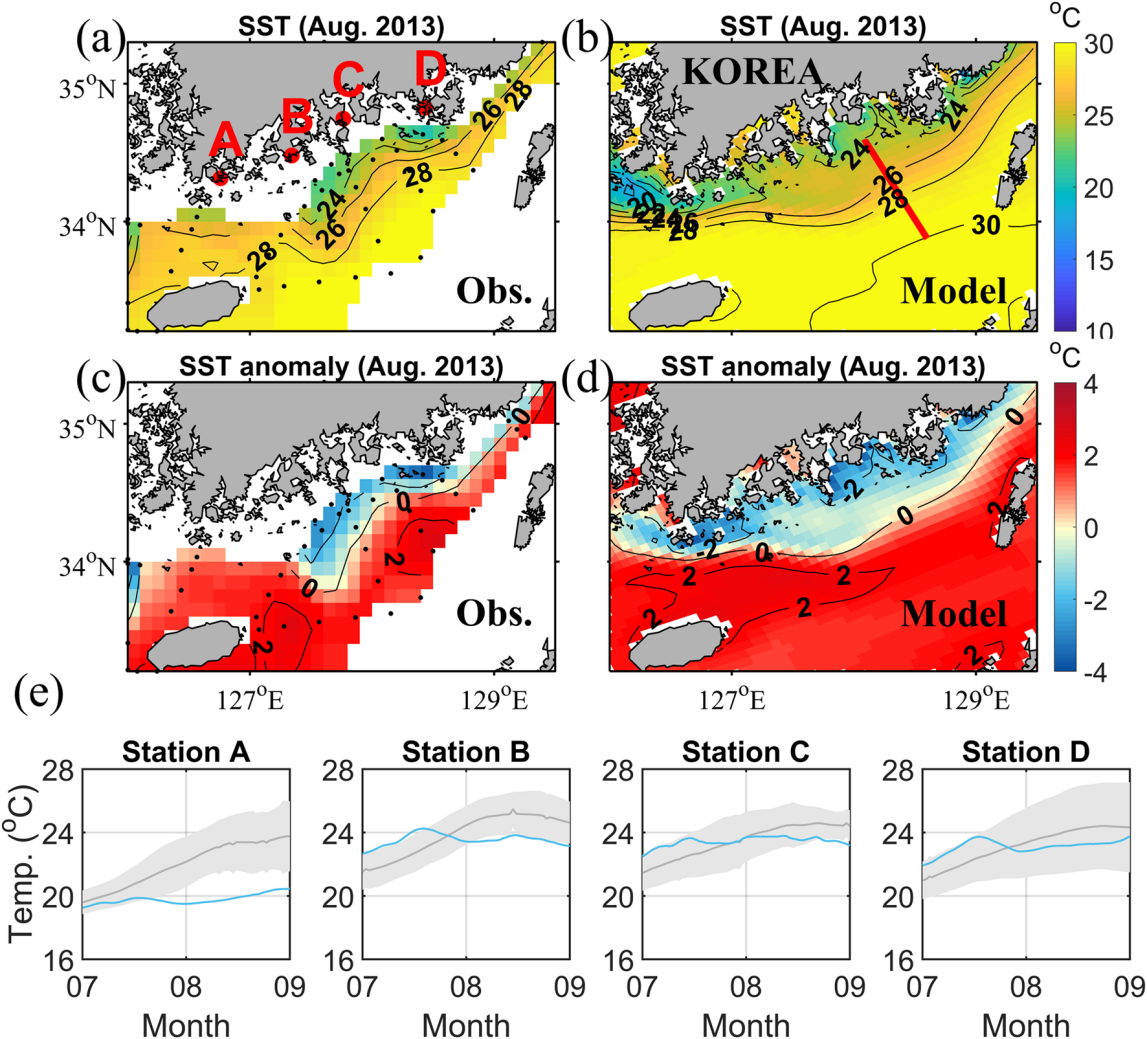
Monthly mean sea surface temperatures (SST) during August 2013 from (**a**) observations (Obs.) and (**b**) model results. The 10 years SST anomaly during August 2013 from (**c**) observations and (**d**) model results. (**e**) Time series of the 2-week running mean SST during 2013 (blue) and the 10 years mean with standard deviation (grey shadow) at tidal stations A–D (red dots in **a**). Black dots in (**a**) and (**c**) indicate the observation stations. Map was generated using M_Map mapping toolbox v1.4 h (https://www.eoas.ubc.ca/~rich/map.html) written for MATLAB.

Unprecedented coastal upwelling was reported in various observations during August 2013. The sea surface temperature (SST) in the coastal region was 2 °C lower than the climatic SST (10 years mean) in the coastal region, whereas the offshore SST was 2 °C higher due to a hot summer in 2013 (Fig. 1c). The cold SST in the coastal region persisted for more than a month after the upwelling-favourable wind weakened (Fig. 1e). The upwelling had serious impacts on the fish farms in this area.

In this study, observational data analyses and numerical modelling were performed to investigate the reason for the unprecedented coastal upwelling, as well as why it was sustained for such an extended period on the southern coast of the Korean Peninsula during the summer of 2013.

## Results

### Unprecedented coastal upwelling

The SST and its anomaly during August 2013 from observational data and model results are shown in Fig. 1. The anomaly was calculated from the 10 years mean SST value (2006–2015). The water temperature near the coastal region was ~ 5 °C lower than the offshore water temperature commonly observed in both observational and modelled data (Fig. 1a, b). This relatively large temperature difference between the coastal and offshore regions was unprecedented. The SST anomaly was remarkable because the water temperature in the coastal region was 2 °C lower than the climatic SST, whereas the offshore water temperature was 2 °C higher (Fig. 1c, d). The positive offshore anomaly was the result of a hot summer in 2013. Despite the positive offshore anomaly, the negative coastal anomaly suggests that there was active coastal upwelling during the summer of 2013. Time series of temperature data from four tidal stations along the coast are shown in Fig. 1e. The 2-week running mean SST during 2013 was similar to, or higher than, the 10 years mean for July, but was lower than the 10 years mean at all stations during August. This also implies that there was strong coastal upwelling during August.

**Figure 2:**
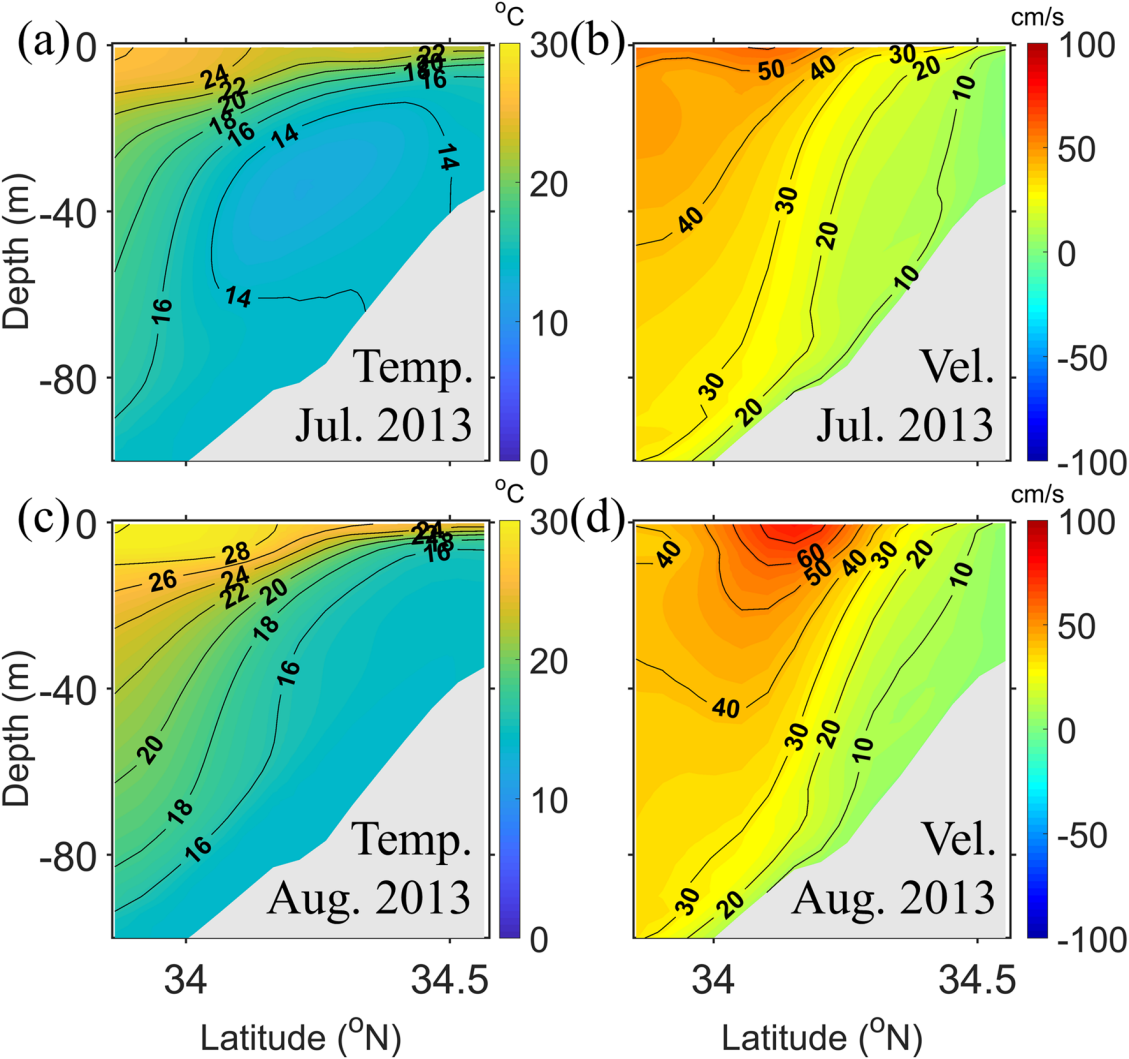
Model calculated cross-shore sections of the monthly mean temperatures (Temp., left) and alongshore velocities (Vel., right) during July (**a**, **b**) and August (**c**, **d**) along the red line in Fig. 1b.

Monthly mean vertical cross sections along the red line in Fig. 1b are shown in Fig. 2. The temperature sections (Fig. 2a, c) show that the isotherms rose in the coastal region, indicating that upwelling occurred during both July and August. The slope of the isotherms in August was steeper than in July. The alongshore velocity sections (Fig. 2b, d) demonstrate that the surface alongshore velocity was higher in August than in July, while the bottom velocity was similarly weak. This suggests that the upwelling was stronger, and the vertical velocity shear was larger in August than in July. The increased vertical velocity shear induced a dynamic uplift of the isotherms that resulted from the geostrophic adjustment between the upper and lower layers in August.

### Momentum balances

**Figure 3:**
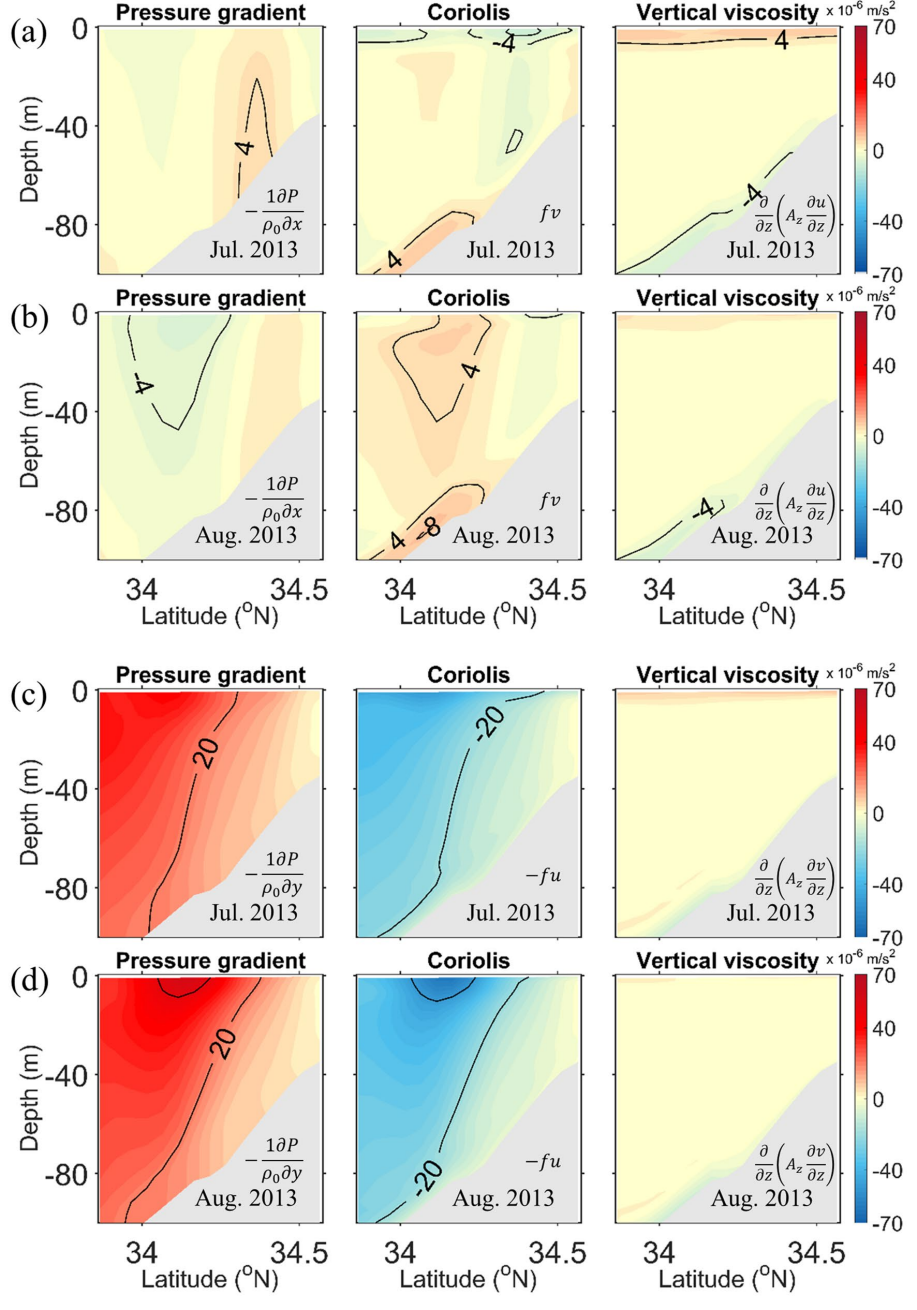
Monthly means of the alongshore momentum balance terms from the model results for (**a**) July and (**b**) August along the red line in Fig. 1b. Monthly means of the cross-shore momentum balance terms for (**c**) July and (**d**) August along the red line in Fig. 1b.

Monthly mean alongshore and cross-shore momentum balances in the cross section along the red line in Fig. 1b were analysed to investigate the cause of the upwelling (Fig. 3). Figure 3a, b illustrate the alongshore momentum terms for July and August, respectively. In July, the vertical viscosity at the surface was balanced with the Coriolis force, which suggests that Ekman transport was induced by the surface alongshore wind-stress. However, the vertical viscosity at the surface was very small in August, compared to July. The Coriolis force and the vertical viscosity at the bottom were balanced in both July and August, indicating the presence of a bottom Ekman layer. The magnitudes of the bottom Ekman layer were similar in July and August. The PGF was balanced with the Coriolis force in the interior region in both July and August.

The PGF and the Coriolis force were remarkable in the cross-shore momentum balance in July and August (Fig. 3c, d). The barotropic pressure gradient induced by the surface slope was larger in August than in July. The pressure gradient in the lower layer decreased due to the baroclinic pressure gradient caused by the dynamic uplift, which resulted in a slow current in the lower layer. The vertical viscosity in the cross-shore momentum balance was relatively small during both months.

A time series of the momentum balance in the coastal and offshore regions (red dots in Supplementary Fig. S1) show the evolution of the momentum balance after the weakening of the wind stress. The PGF and Coriolis force increased gradually in the cross-shore direction, while the vertical viscosity at the surface decreased rapidly in the alongshore direction, according to the weakening of the wind stress in early August (Supplementary Figs. S2 and S3).

### Temporal variations in the upwelling index and causes that drive upwelling

To examine the relationship between the upwelling strength and its possible causes, the upwelling index (UI), the wind-driven upwelling transport, the Ekman pumping transport, the slope of the interface representing dynamic uplift, and the sea level difference between the coastal and offshore regions were calculated.

**Figure 4:**
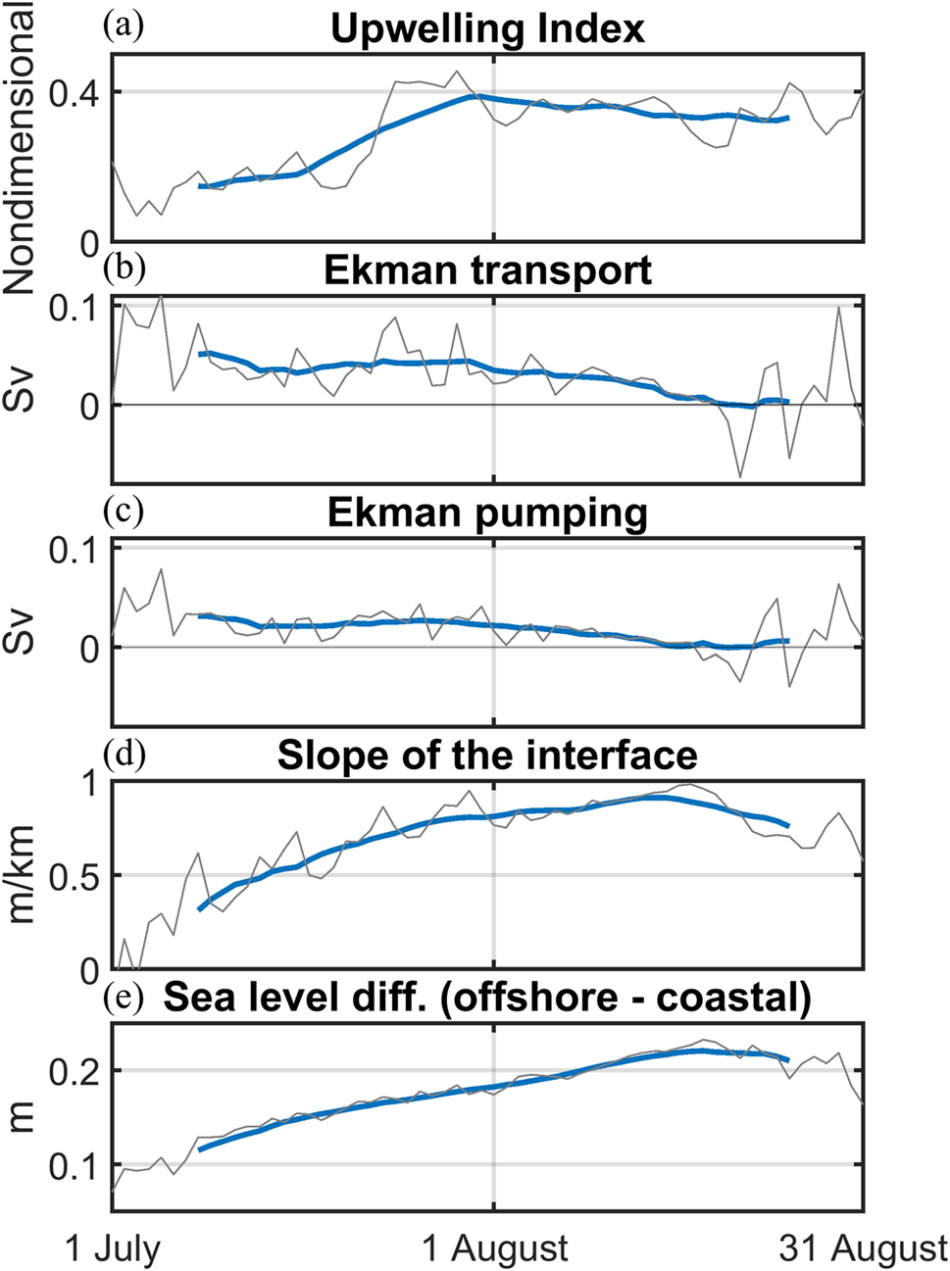
Time series of (**a**) the upwelling index, (**b**) Ekman transport, (**c**) Ekman pumping transport, (**d**) the slope of the interface, and (**e**) the sea level differences between the coastal and offshore regions from 1 July to 31 August, 2013. The grey and blue lines indicate the daily mean and the 2-week running mean, respectively.

The UI increased rapidly in mid-July and reached its maximum in late July (Fig. 4a). This corresponded with the temporal variations in temperature observed at the tidal stations (Fig. 1e). Ekman transport and Ekman pumping were significantly large during July, which might have been crucial causes of coastal upwelling (Fig. 4b, c). However, both decreased dramatically as the wind speed decreased during August. It is obvious that the coastal upwelling in July was induced by the wind. However, a high UI persisted until the end of August despite the collapse of the upwelling-favourable wind. The slope of the interface increased during July and continued to have high values during August, as did the UI (Fig. 4d). This implies that the persistence of the coastal upwelling was closely related to the persistent dynamic uplift. The large sea level differences between the coastal and offshore regions continued after the plunge in the upwelling-favourable wind, as did the slope of the interface (Fig. 4e).

## Discussion and conclusions

**Figure 5:**
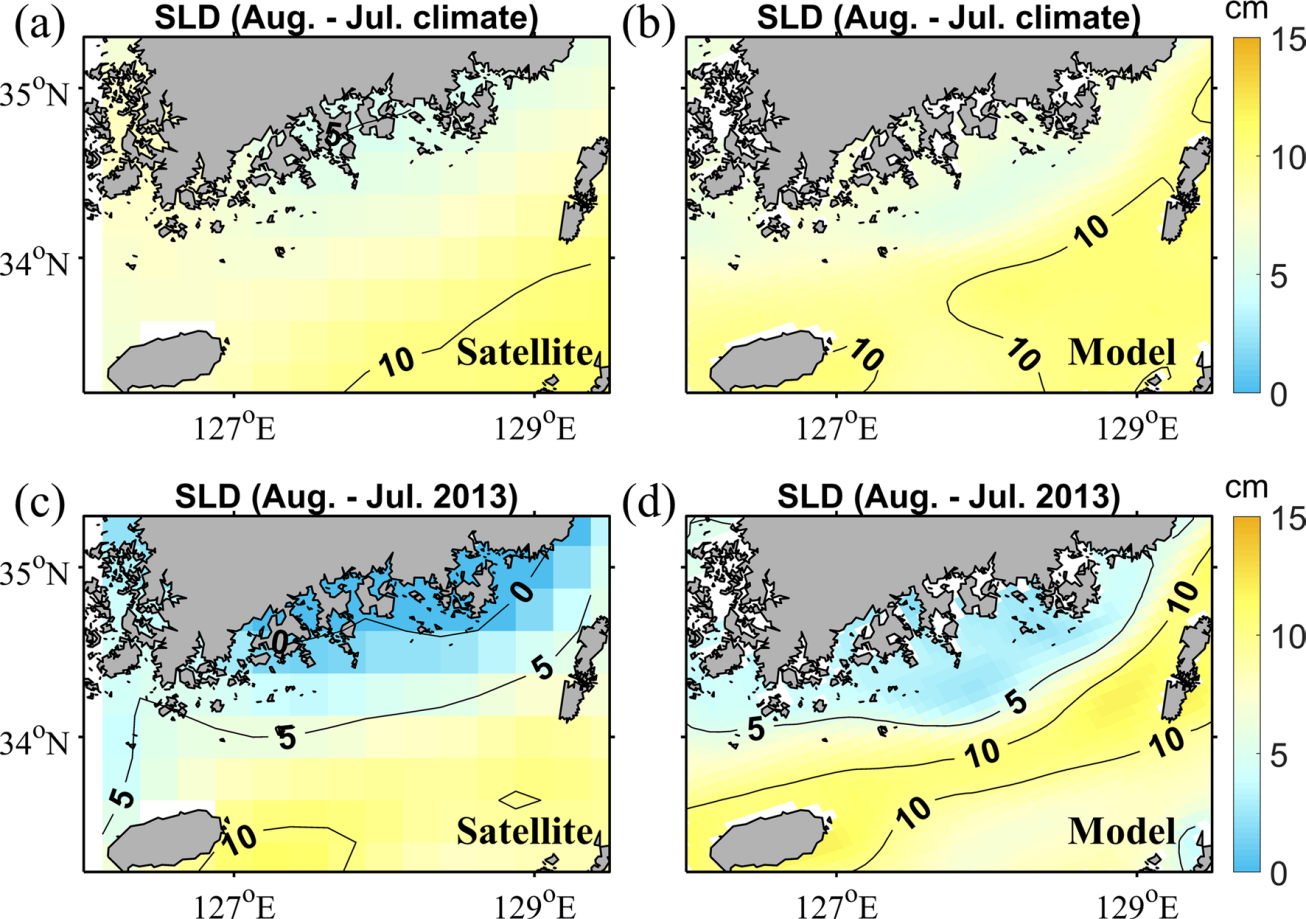
Sea level differences (SLD) between July and August 2013 as determined using the 10 years mean (2006–2015) from (**a**) satellite observations and (**b**) model results. Sea level differences between July and August 2013 from (**c**) satellite observations and (**d**) model results. Map was generated using M_Map mapping toolbox v1.4 h (https://www.eoas.ubc.ca/~rich/map.html) written for MATLAB.

It is known that most coastal upwelling events relax a few days after the upwelling-favourable winds weaken^12,13^. However, coastal upwelling may continue, due to geostrophic equilibrium, for an extended period^14^. The sea level decreases significantly in upwelling regions, which increases the cross-shore pressure gradient due to the sea level difference^2,15^. The monthly mean sea level differences between July and August were determined from satellite data and from the model results (Fig. 5). The sea level differences in 2013 in the coastal region were much smaller than those determined using the 10 years mean data, whereas the sea level differences in the offshore region were almost the same. This suggests that the sea level in the coastal region during August 2013 was lower than that of the 10 years mean. The sea level in the upwelling area decreased during a period of upwelling-favourable winds from early July to late July. The decreased sea level was maintained after the weakening of the upwelling favourable wind in August. The decreased sea level in the upwelling region may have maintained the cross-shore pressure gradient in August.

In the southern coastal region of the Korean Peninsula, the alongshore current flows eastward throughout the year^8,9^. When wind-driven upwelling occurred in 2013, the intensified cross-shore pressure gradient strengthened the surface alongshore current. The increased surface velocity led to a dynamic uplift due to the resulting geostrophic adjustment. The upwelled cold water that resulted from the dynamic uplift lowered the coastal steric sea level. The decreased sea level in the coastal area intensified the cross-shore barotropic pressure gradient, which induced a strong geostrophic current. However, the current in the lower layer decreased due to the baroclinic pressure gradient caused by the dynamic uplift. The intensified surface alongshore current subsequently produced a dynamic uplift due to the geostrophic adjustment.

Unprecedented coastal upwelling in the southern coastal region of the Korean Peninsula was reported via observational data measured during the summer of 2013. Observational data and model results obtained in this study show that the upwelling occurred due to upwelling-favourable wind during July. The upwelling persisted until the end of August, despite a weakening of the upwelling-favourable wind. After a plunge in the upwelling-favourable wind, a positive feedback between the dynamic uplift and geostrophic adjustment maintained the coastal upwelling for a month. The coastal upwelling that was driven by the upwelling-favourable wind lowered the sea level in the coastal region, which enhanced the surface alongshore current due to the cross-shore sea level difference. The strong surface alongshore current maintained the dynamic uplift of deep, cold water in the coastal region due to the geostrophic equilibrium. Additional research is necessary to provide a dynamic explanation of the shut-down process of the upwelling in the study area.

## Methods

### Temperature observations

Two observational temperature datasets from 2006 to 2015 were used for this study. One was tidal station data that consisted of continuously observed data at 1 h intervals, which was obtained from the Korea Hydrographic and Oceanographic Agency (https://www.khoa.go.kr/oceangrid/koofs/kor/observation/obs_real.do). The other dataset was serial oceanographic observations obtained from the National Institute of Fisheries Science (https://www.nifs.go.kr/kodc/index.kodc). The serial oceanographic data have been routinely observed on a bimonthly basis at standard ocean depths around the Korean Peninsula.

### Absolute dynamic topography

Satellite derived absolute dynamic topography (ADT) from 2006–2015 were obtained from the Copernicus Marine Environment Monitoring Service (CMEMS, https://marine.copernicus.eu/services-portfolio/access-to-products/?option=com_csw&view=details&product_id=SEALEVEL_GLO_PHY_L4_REP_OBSERVATIONS_008_047).

### ROMS model setup

The numerical model used in this study was the Regional Ocean Modeling System (ROMS)^16^, which is a free-surface, split-explicit, and hydrostatic ocean model that is characterised by a terrain-following curvilinear system. The model domain included the Yellow Sea, the East/Japan Sea, and part of the East China Sea. The model grid had a resolution of 6–8 km horizontally and 40 vertical layers. ETOPO1^17^ (https://doi.org/10.7289/V5C8276M) and KorBathy30s^18^ data were used for the bottom topography, with a minimum depth of 7 m. The initial temperature and salinity data were obtained from the World Ocean Atlas 2018 (WOA 2018)^19,20^. HYCOM GOFS 3.0 reanalysis and analysis data were adopted for the open boundary (https://www.hycom.org/dataserver/gofs-3pt0). The 6 hourly data from the European Centre for Medium-Range Weather Forecasts (ECMWF) ERA5 reanalysis were used for the surface forcing, including temperature, wind, air pressure, and relative humidity^21^ (https://doi.org/10.24381/cds.bd0915c6). Daily mean values were used for solar radiation and precipitation. A bulk-flux formulation was used for calculating the surface flux^22^. Tidal forcing was applied along the open boundaries using ten major tidal components to include the tidal mixing effect that results from tidal elevation and tidal currents^23^ (https://volkov.oce.orst.edu/tides/TPXO7.2.html). Discharges from 12 rivers were also included. Monthly mean river discharges at the Datong gauging station were used for the Changjiang River (https://www.cjh.com.cn/sqindex.html). River discharges for the other 11 rivers were obtained from the Global River Discharge Database^24^ (https://doi.org/10.3334/ORNLDAAC/199). Vertical mixing was calculated using the K-profile parameterization mixing scheme^25^. Chapman, Flather, and clamped boundary conditions were used for the free-surface, barotropic, and baroclinic momentums, respectively^26^. The horizontal viscosity coefficient was set to 100 m^2^/s. The model was integrated for 15 years (from 2001 to 2015) after a 10 years spin-up run. The model results from 2006 onward were analysed.

### ROMS momentum balance analysis

The momentum balance terms were calculated from the model results following Eqs. (1) and (2) by neglecting the acceleration, advection, diffusion, and horizontal viscosity terms (Fig. 3)

1 $$\underbrace {{\frac{\partial u}{{\partial t}}}}_{Acceleration} = - \underbrace {{\frac{1}{\rho }\frac{\partial P}{{\partial x}}}}_{Pressure gradient} + \underbrace {fv}_{Coriolis} + \underbrace {{\frac{\partial }{\partial z}\left( {A_{z} \frac{\partial u}{{\partial z}}} \right)}}_{Vertical viscosity},$$

2 $$\underbrace {{\frac{\partial v}{{\partial t}}}}_{Acceleration} = - \underbrace {{\frac{1}{\rho }\frac{\partial P}{{\partial y}}}}_{Pressure gradient} - \underbrace {fu}_{Coriolis} + \underbrace {{\frac{\partial }{\partial z}\left( {A_{z} \frac{\partial v}{{\partial z}}} \right)}}_{Vertical viscosity},$$

where *u* and *v* are the alongshore and the cross-shore velocity components, respectively, *P* is the pressure, $\rho$ is the density of seawater, $f$ is the Coriolis parameter, and ${A}_{z}$ is the vertical eddy viscosity.

### Upwelling index (UI) calculation

The UI was calculated using Eq. (3), which is a modified form of the UI suggested by Demarcq and Faure^27^ based on the SST (Fig. 4a)

3 $$\mathrm{U}\mathrm{I}=\frac{{Temp}_{offshore surface}-{Temp}_{coastal surface}}{{Temp}_{offshore surface}-{Temp}_{coastal bottom}}.$$

The modelled daily temperatures were used for the calculation. The surface temperature 150 km from the coast was chosen as the offshore temperature in each grid. UIs of 0 and 1 indicate no upwelling and the maximum upwelling, respectively. The UI was averaged along the coastal grids for each day.

### Wind-driven upwelling transport

The wind-driven upwelling transport includes Ekman transport and Ekman pumping^28^ (Fig. 4b, c). The Ekman transport in each coastal grid, *M* (m^3^/s per meter of coast), was calculated after Smith^29^, as expressed by Eq. (4):

4 $$M=\frac{\overrightarrow{\tau }\bullet \widehat{t}}{\rho f},$$

where $\overrightarrow{\tau }$ is the wind-stress vector, $\widehat{t}$ is a unit vector tangent to the coastline, $\rho$ is the density of seawater, and $f$ is a Coriolis parameter. The 6 h wind data were used to calculate the wind-driven upwelling. Ekman transport was integrated along the coastal grid.

The Ekman pumping velocity, *w* (m/s), was calculated after Smith^29^, as defined in Eq. (5):

5 $$w=\widehat{k}\bullet \nabla \times \frac{\overrightarrow{\tau }}{\rho f},$$

where $\widehat{k}$ is a unit vector in the local vertical direction. Ekman pumping velocities were integrated 100 km offshore from the coastal grid to calculate the Ekman pumping transport.

### Slope of the interface

Assuming the geostrophic balance between two layers, the slope of the interface, ${\partial h}_{2}/\partial y (\mathrm{m}/\mathrm{k}\mathrm{m})$, can be calculated from the daily mean of the model results (Fig. 4d) using Eq. (6):

6 $$\frac{{\partial h}_{2}}{\partial y}=\frac{f({u}_{1}{\rho }_{1}-{u}_{2}{\rho }_{2})}{g({\rho }_{2}-{\rho }_{1})},$$

where ${\rho }_{1}$ is the density of the upper layer, ${\rho }_{2}$ is the density of the lower layer, ${u}_{1}$ is the alongshore velocity in the upper layer, and ${u}_{2}$ is the alongshore velocity in the lower layer. The selected density for the interface between the two layers was 1,024 kg/m^3^. The slope of the interface was averaged along the coast after the calculations using the model cross-shore vertical sections from the coastal grid to the grid 100 km offshore.

### Sea level differences between the coastal and offshore regions

The sea level differences between the coastal and offshore regions were calculated from the daily mean model results (Fig. 4e). The sea level 100 km offshore was chosen as the offshore sea level. The sea level difference between the coastal and the offshore grids was spatially averaged along the coast.

## Supplementary information

Supplementary information 

## Data Availability

All data used in this research may be downloaded from the links provided in the methods section. The model results are freely available upon request from the corresponding author.
