# Supplementary material for: Persistence of coastal upwelling after a plunge in upwelling-favourable wind
Source: Sci Rep. 2020 Jul 20;10:11938. doi: 10.1038/s41598-020-67785-x (PMC7371861; doi:10.1038/s41598-020-67785-x)
Supplement: Supplementary file 1 — Supplementary information [file 41598_2020_67785_MOESM1_ESM.pdf]

Supplementary information for

Persistence of coastal upwelling after a plunge in upwelling-favourable wind

Jihun Jung and Yang-Ki Cho

School of Earth and Environmental Sciences/ Research Institute of Oceanography, Seoul National University, Seoul 151-742, Republic of Korea

Corresponding author: Yang-Ki Cho (choyk@snu.ac.kr)

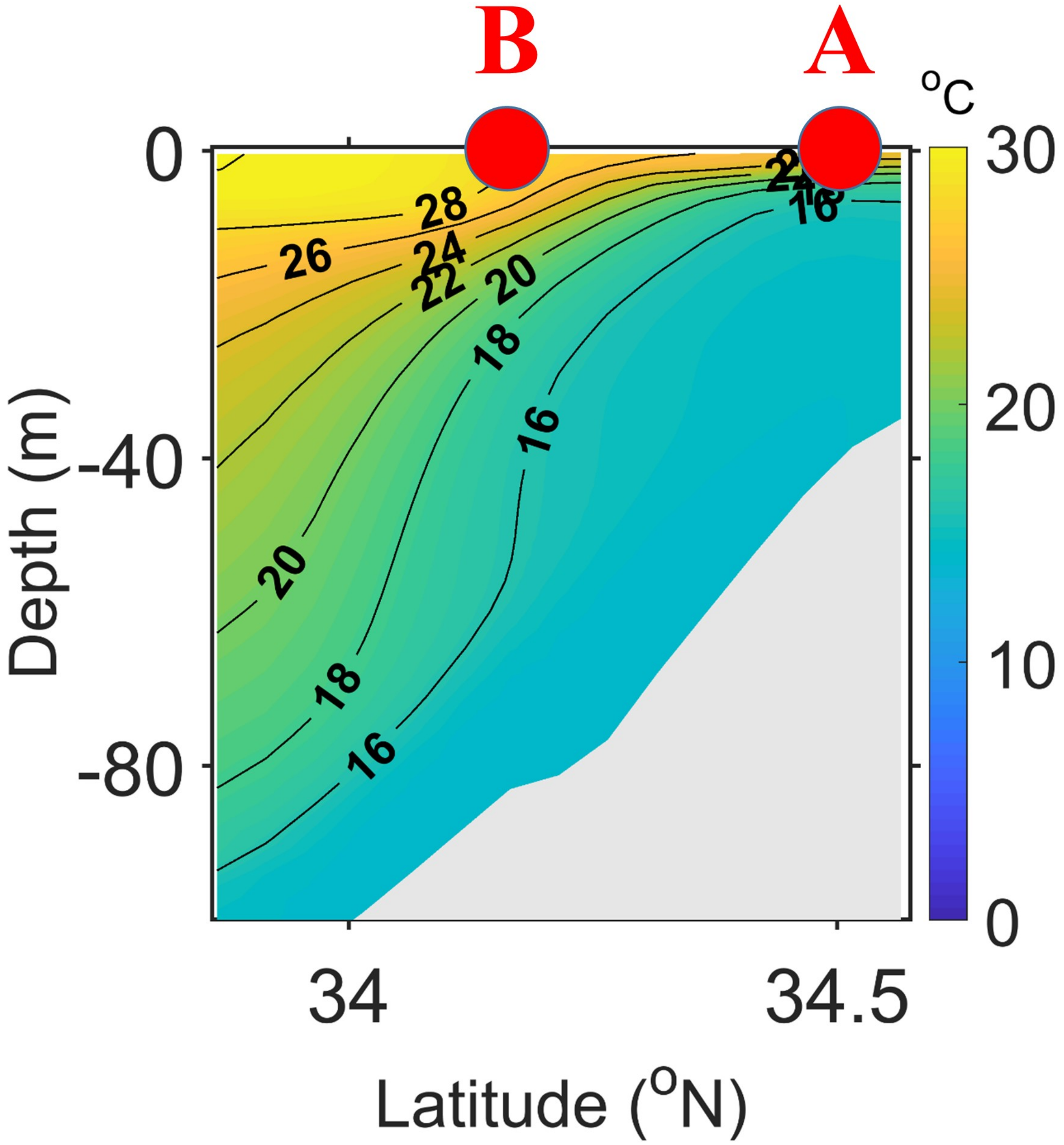

Supplementary Figure S1. Model calculated cross-shore section of the August monthly mean temperatures along the red line in Fig. 1b.

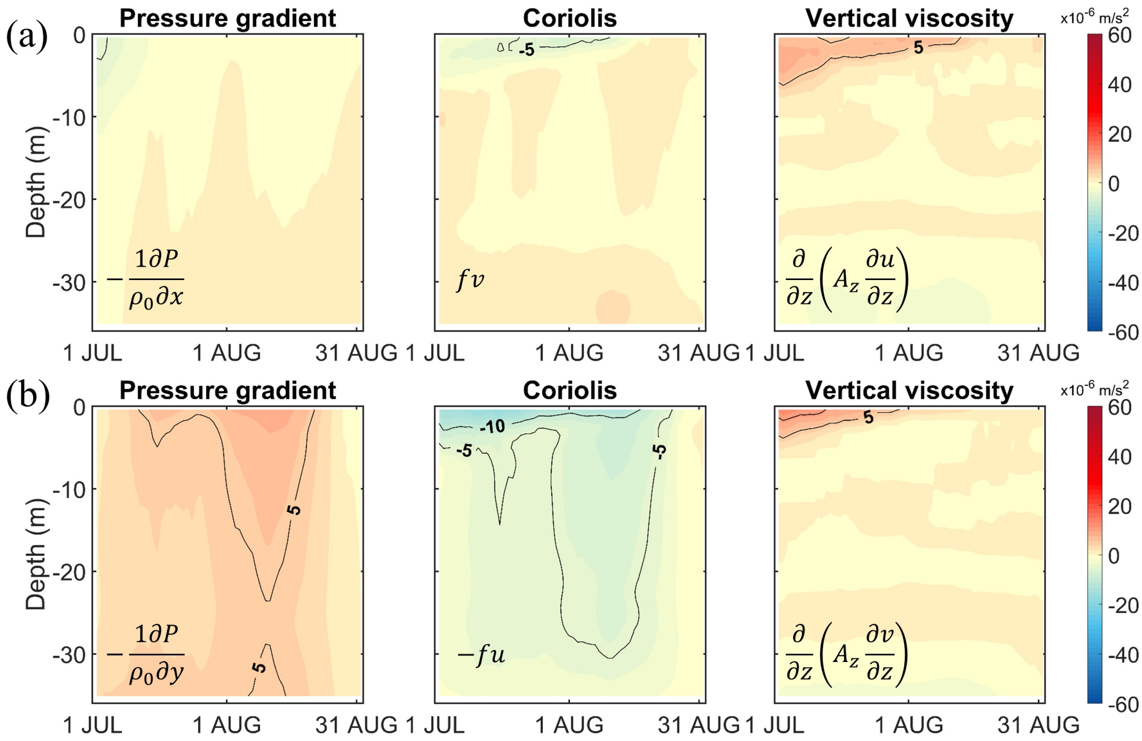

Supplementary Figure S2. Time series of (a) alongshore momentum balance terms and (b) cross-shore momentum balance terms at the red dot A in Fig. S1. All terms are filtered by a 2-week running mean of the daily means.

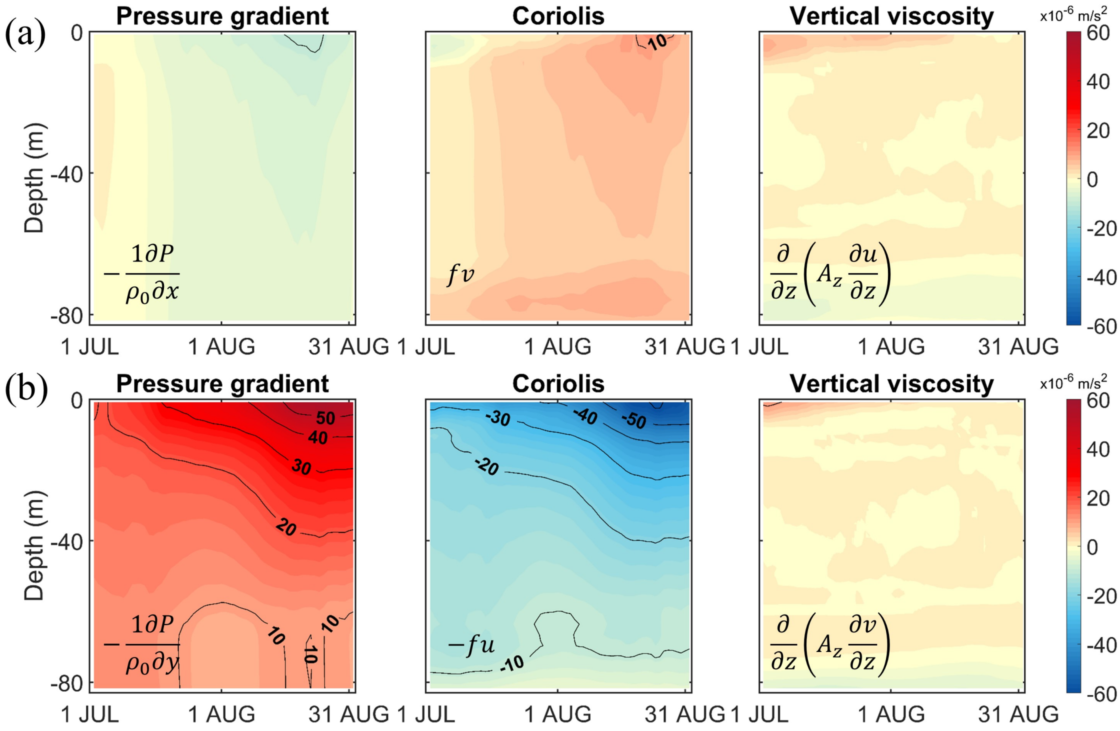

Supplementary Figure S3. Time series of (a) alongshore momentum balance terms and (b) cross-shore momentum balance terms at the red dot B in Fig. S1. All terms are filtered by a 2-week running mean of the daily means.
